# Supplementary material for: Temporal trends in the incidence and case severity of COVID-19 cases among the Syrian refugees in Azraq camp in Jordan: A retrospective observational study
Source: PLoS Negl Trop Dis. 2025 Feb 19;19(2):e0012875. doi: 10.1371/journal.pntd.0012875 (PMC11867313; doi:10.1371/journal.pntd.0012875)
Supplement: Attachment — Submitted filename: Response to Reviewers.docx [file pntd.0012875.s002.docx]

Dr. Shaden Kamhawi
Editor-in-Chief
PLOS Neglected Tropical Diseases

November 28, 2024‎

Dear Professor Kamhawi,‎

RE: " Temporal trends in the incidence and case severity of COVID-19 cases among the Syrian ‎refugees in Azraq camp in Jordan: a retrospective observational study‎"‎

Thank you for the opportunity to have our work considered by PLOS Neglected Tropical Diseases. On ‎behalf of the investigators, we wish to thank the editors and reviewers for their valued ‎contributions and provide the following responses to their appraisals.‎

In response to reviewers’ comments by section

**Methods**

**Comment 1:**

Reviewer #1: The article aimed to capture epidemiological characteristics, evaluation of policies enforced and vaccine, risk and outcome of COVID-19 among the Syrian refugees in Azraq camp, Jordan. Collected data include socio-demographic characteristics, comorbidities, COVID-19 test history and results, contact history and vaccination.

Some conditions may need to be considered, such as recurrent cases, population dynamics (in addition to underregistration, other things like inflow/outflow of the population that were managed by external authorities or effect of birth/death by other causes) and underreporting. It sounded a bit strange without mentioning these points.

**Response 1:**

We thank the reviewer for this important comment. We acknowledge the importance of reporting these conditions such as population dynamics and underreporting. However, the collected data for this study was limited by confidentiality policies enforced by the managing authorities, preventing access to detailed information on these conditions. Despite these constraints, we have addressed population inflow in the manuscript, noting that 9% of cases were detected upon entry to the camp during mandatory COVID-19 testing at entry points. Additionally, underreporting and other unmeasured factors have been explicitly acknowledged in the limitations section as potential influences on the observed trends as follow:

*“Future research should aim to address these gaps by fostering collaboration with relevant stakeholders, including camp authorities (i.e. UNHCR) and international organizations, to gain access to comprehensive data on population dynamics, including inflow and outflow, birth and death rates, and other demographic changes”.*

**Comment 2:**

Especially, recurrent cases are important as they would be correlated to the incidence, and I would recommend to consider the correlation of recurrent cases in all analysis: for example, risk factor analysis may be influenced by the correlation of recurrent cases as it means "double-counted". All results may be changed due to this point. If the author already considered this point, analyses method might be considered and selected correspondingly as well as well-mentioned in the text.

**Response 2:**

We agree with the reviewer. Recurrent cases are indeed critical to consider, and we have included all individuals who tested positive for COVID-19, including those with multiple positive tests over time. These cases were retained because our study focuses on the incidence rates across different waves of infection, rather than analyzing individual-level recurrence patterns.

In our approach, recurrent cases were treated as "new cases" when they occurred in different waves, consistent with the evolving nature of COVID-19. Each wave of infection was driven by distinct variants with differing characteristics, transmissibility, and immune escape potential, which effectively created new epidemiological contexts. Retaining recurrent cases allowed us to capture these dynamics and provided a broader perspective on the virus's progression and the impact of interventions over time. We further clarified using them as new current cases in the text as follow:

*“Recurrent cases were retained, as they may provide a deeper understanding of the incidence across different waves and offer a broader perspective on the progression of the virus over time.”*

**Comment 3:**

Some comments in the analysis part.
First, comparison test in Table 3 is blurry. Which test were made in comparing which proportion between subgroups?

**Response 3:**

We thank the reviewer for pointing out this issue. We used chi-square tests to compare proportions between subgroups in Table 3, as these tests are suitable for categorical data. This was stated in the statistical analysis section (line 162). However, we acknowledge that this may not have been immediately clear to readers.

To address this, we have added a detailed footnote to Table 3 specifying that chi-square tests were used to assess differences in proportions across subgroups such as age, gender, comorbidities, and other variables. Additionally, we revised the statistical analysis section to re-emphasize this point, ensuring that the methods are fully transparent and easily understood.

**Comment 4:**

The unit of incidence varies across analysis. I would say all analysis can use a unit of 1000 subpopulation to describe incidence.

**Response 4:**

We appreciate the reviewer’s suggestion regarding standardizing the unit of incidence. In our analysis, we deliberately used different units of incidence rates based on the context of the comparison. When comparing the camp incidence with the national figures, we used rates per 100,000 population, as this is the standard unit in epidemiological studies and national-level reporting. This approach ensures that the results are directly comparable to national statistics, facilitating broader relevance and alignment with global reporting standards.

For camp-specific analyses, we used incidence rates per 1,000 individuals to provide a more precise and interpretable measure of disease dynamics within the smaller population of approximately 44,000 residents. This unit was chosen to reflect the specific epidemiological characteristics of the camp while avoiding potential misinterpretations that could arise from using a larger denominator in a small community.

**Comment 5:**

Also, how did you estimate and hypothesize the effect of each month.

**Response 5:**

We thank the reviewer for this important question. The effect of each month was estimated by analyzing the incidence trends over the two-year study period, which included distinct COVID-19 waves characterized by sharp rises and subsequent declines in cases. This approach allowed us to capture the dynamic temporal patterns of disease transmission influenced by factors such as the emergence of new variants, changes in public health measures, and vaccination campaigns.

We hypothesized that monthly trends would reflect the progression of the pandemic, with variations driven by factors like variant transmissibility, immunity levels, and public health interventions. To analyze these trends, we treated the calendar month as a continuous variable in a Poisson regression model, assuming that the monthly count of cases followed a Poisson distribution. This approach is standard for evaluating temporal changes in incidence rates, particularly in settings with relatively stable population demographics, as was the case in the Azraq camp during the study period.

The decision to analyze trends by month aligns with methodologies used in epidemiological studies and incidence reporting by global health organizations, such as the WHO and CDC.

<https://www.who.int/europe/news/item/02-08-2024-amid-a-summer-wave-of-covid-19--a-new-who-europe-study-confirms-the-lifesaving-impact-of-vaccines>

<https://www.cdc.gov/ncird/whats-new/covid-19-can-surge-throughout-the-year.html>

**Comment 6:**

Why and what did you assume the linear trend of change across months to do monthly change analysis? Please mention in the main text.

**Response 6:**

We thank the reviewer for this question. We did not assume a linear trend in the monthly change analysis. Instead, we used a Poisson regression model, which is well-suited for analyzing count data, such as the number of positive COVID-19 cases per month. This approach assumes that the outcome (positive cases) follows a Poisson distribution, which is appropriate for rare events or count data observed over time.

The choice of Poisson regression allowed us to capture temporal changes in incidence rates without imposing a linear trend assumption. Instead, the model estimated the relative change in the incidence rate for each calendar month while accounting for the count nature of the data. This approach provides a more flexible and accurate representation of how incidence rates evolved over the study period, especially in the context of distinct COVID-19 waves, which are inherently non-linear.

**Comment 7:**

Furthermore, which test or regression did you use for risk facrtor analysis for calculating adjusted odds ratio? Maybe logistic regression?

**Response 7:**

We thank the reviewer for this question. For the analysis of risk factors, we used univariable and multivariable multilevel logistic random-effects regression models, with clustering by village. This approach is explicitly described in the statistical analysis section of the manuscript.

The multilevel (hierarchical) logistic regression model was chosen because our data included individuals nested within villages, creating a clustered structure. By incorporating random effects for villages, this method accounted for potential within-cluster correlation, thus improving the accuracy and robustness of the estimated aORs. This was particularly important given the potential variations in healthcare access, detection methods, and public health interventions among different villages in the camp.

**Comment 8:**

Moreover, how did you determine final model to include potential confounders? how does the effect of each confounders?

**Response 8:**

We appreciate the reviewer’s question. To ensure a comprehensive assessment of potential confounders, we included all available variables in the final model, regardless of their statistical significance in the univariable analysis. This approach was chosen to minimize the risk of residual confounding and to provide a more robust estimation of aORs for the primary risk factors. In the final model, variables included age, gender, comorbidities, vaccination status, and calendar month. While gender was not statistically significant in predicting hospitalization (Table 4), it was retained in the model to control for any potential confounding effects it might have on the relationship between other covariates and the outcome. This decision aligns with best practices in epidemiological analysis, where theoretically important variables are often included to account for their potential influence, even if their direct association with the outcome is not statistically significant.

**Comment 9:**

Reviewer #2: Methods are clearly articulated, however, below needs to be addressed -

134: This is a retrospective study. But data were collected “prospectively”. This is confusing. Please clarify.

**Response 9:**

We appreciate the reviewer’s observation regarding the terminology used to describe the data collection process. To clarify, the data were collected at the time of occurrence by the healthcare teams in the camp as part of routine COVID-19 case management and surveillance efforts. This collection process was prospective in nature, as it occurred contemporaneously with the events. However, our study analyzed the dataset retrospectively, as the data were reviewed and analyzed after the study period had concluded. We understand that the use of the term “prospectively” in the manuscript could create confusion regarding the study design. To eliminate ambiguity, we deleted the term “prospectively” from the text.

**Comment 10:**

141: What data collection tool or instrument was used. It should be annexed.

**Response 10:**

We thank the reviewer for this important comment. As described in the text, data collection was conducted through face-to-face and phone interviews as part of a community case management program implemented by the IMC. However, the specific data collection tool or instrument used by the organization was not made available to us due to confidentiality policies. As a result, we are unable to annex the data collection instrument to the manuscript.

Reviewer #3: (No Response)

**Results**

**Comment 11:**

Reviewer #1: I would assume that the monthly incidence IRR change was calculated using Poission regression, with the calendar month taken into the analysis as continuous variables.

If so, why would you assume linearity trend of risk of COVID-19 per each month? As you present some results, could you please provide some interpretation, such as what the increasing/decreasing % mean? Do you think it is really the case, as it is obvious that the cases are recorded like waves?

**Response 11:**

To clarify, we did not assume a linear trend in the monthly incidence of COVID-19 cases. Instead, we employed a Poisson regression model to analyze the count data, which is appropriate for modeling discrete outcomes such as the number of COVID-19 cases. As described in the manuscript (lines 167-169), the calendar month was included as a continuous variable in the model to estimate the average change in incidence rates over time. This approach does not impose a linear trend assumption but instead quantifies the relative change in incidence rates for each calendar month.

The IRR reflects the average monthly change in incidence rates across the study period. When the IRR exceeds 1.00, it indicates an increase in the incidence rate, while an IRR below 1.00 reflects a decrease. This average change represents the overall trend observed during the study period, capturing the progression of incidence rates while accounting for wave-like patterns. It is important to note that the primary outcome was the count of positive cases, and the IRR provides a summarized measure of how these counts changed over time.

While cases followed wave-like patterns due to the emergence of new variants and changes in public health interventions, the use of Poisson regression allowed us to estimate these trends without assuming uniformity or linearity. The observed changes in IRR are consistent with global epidemiological patterns, where surges and declines in cases correspond to specific pandemic waves. This finding is further supported by visual representations (e.g., Figures 1 and 2), which show the temporal dynamics of incidence rates.

**Comment 12:**

And it might be better to explain IRR more: some IRR compares between cases in Azraq camp, while others between one month and the next. Maybe its better to determine in the other way as it might be confusing using the term "IRR" in a different manner.

**Response 12:**

We thank the reviewer for their suggestion regarding the potential confusion in the use of IRR. To clarify, the plotted IRR in Figure 1 represents the relative difference in the incidence rates between Azraq camp and the national level for each month, rather than changes between consecutive months. This measure allows us to compare the monthly incidence trends in the camp to the broader national context, highlighting differences in disease dynamics between the two populations.

Elsewhere in the manuscript, the IRR was estimated using Poisson regression to quantify the average monthly change in incidence rates across the study period, rather than comparisons between consecutive months. This approach provides an overall measure of temporal trends while accounting for the wave-like nature of the data.

To avoid confusion, we have revised the text in the methods and results sections to explicitly describe the context and meaning of IRR in each instance. o avoid confusion, we have revised the text in the methods and results sections to explicitly describe the context and meaning of IRR in each instance.

**Comment 13:**

Adjustment of confounders (age, sex, etc.) were not performed in poisson regression?

**Response 13:**

We thank the reviewer for this important comment. In our analysis, adjustment for age and gender in the Poisson regression model was not performed because these variables were consistent over the study period for the population of the Azraq camp. The demographic composition of the camp, including the distribution of age and gender, did not show significant changes during the study period. As a result, these variables did not act as confounders in the analysis of temporal trends in COVID-19 incidence.

Confounders are variables that are associated with both the exposure and the outcome and can distort the true relationship being examined. In this case, our primary outcome was the incidence rate of COVID-19 cases over time, and the independent variable of interest was the calendar month. Since the age and gender distribution remained stable, they did not introduce variability that could confound the relationship between the calendar month and COVID-19 incidence rates

**Comment 14:**

Also, it would be better to have mapping and show relationship between villages (such as the interaction between villages, providing backgrounds of linkages).

**Response 14:**

Thank you for an insightful point to consider, we clarified this linkage in the study setting as follows:

*“Villages 2,3, and 6 were directly interconnected, whereas village 5 was entirely isolated as a strategy of suppressing the virus.”*

In the discussion section, we expanded on the impact of these strategies by highlighting how the isolation of Village 5 correlated with its higher proportion of cases detected through active surveillance (>60%). This demonstrates that targeted containment measures were promptly implemented by authorities in response to epidemiological findings, effectively managing disease transmission within the camp. the discussion section as follows:

*“Village 5 had reported the largest proportion of cases detected through surveillance (>60%), which justifies the isolation of the village from the other neighboring villages during the pandemic outbreak, indicating that the authorities were closely monitoring the epidemiological situation in the camp and promptly implementing immediate, appropriate actions.”*

While we recognize the value of including a detailed map to illustrate these village interactions, we opted not to include one due to the limited availability of geospatial data and the constraints of the study design. Instead, we have described these relationships in detail in the manuscript to provide readers with a clear understanding of the epidemiological context.

We believe these clarifications adequately address the reviewer’s concern while maintaining the focus of the manuscript. If future studies in similar settings collect geospatial data, mapping village interactions could provide additional insights into the dynamics of disease transmission.

**Comment 16:**

Moreover, interpretation of IRR by home-based isolation strategy (Line 242) requires carefulness: it only indicates some evidence, not strong, so it might be dangerous to say "increased" IRR in this section.

**Response 16:**

Thank you for this comment. We have amended the interpretation as follows:

“Isolation in a public space strategy declined ‎(IRR: 0.981; 95% CI: 0.971-‎‎0.992; p-‎value=0.001)‎ over the study period while home-based isolation tended to show a ‎slight increase (‎IRR: 1.007; 95% CI: 0.999-1.015; p-value=0.087‎).‎

**Comment 17:**

Regarding to risk factor analysis: I think the results between behavior and severity is biased: one possible explanation is that they seek for healthcare as they are more severe. So it provides more value if you could mention such bias and how to control them in the main text. Also in the main text, I think you could conduct chi-squared test to compare the positive rates between subcategories to strengthen the findings.

**Response 17:**

We thank the reviewer for raising this important point. As noted, individuals who sought healthcare were more likely to have severe symptoms, which could result in overrepresentation of severe cases among those detected through healthcare-seeking behavior. To address this, we explicitly acknowledged this bias in the discussion section and provided the following explanation in the main text:

"Those who sought healthcare were more likely to have severe symptoms compared to other modes of detection, which may have contributed to an overrepresentation of severe cases among those tested positive for COVID-19."

To strengthen our analysis, we conducted chi-squared tests to compare positive rates and case severity across subcategories such as demographics, vaccination status, and comorbidities. These results, presented in Table 3, highlight significant associations between these variables and case severity, providing additional robustness to our findings. We ensured that the methodology for these tests was clearly described in the statistical analysis section and have clarified in the table footnotes that chi-squared tests were used for these comparisons.

**Comment 18:**

Finally, please fix minor errors in main text: I think Sep and Oct 2020 not 2022 (Line 185) and aOR for hospitalization among vaccinated group compared to non-vaccinated in multivariable analysis (Line 305; I think table 4 is correct, so text should be modified like 95%CI: 0.38-0.81).

**Response 18:**

- The date error was fixed.

- Line 305 mentioned the cases with comorbidities, not vaccinated cases, so the written data in the text is correct. Kindly see lines 308- 311 for hospitalization among vaccinated group.

**Comment 19:**

Reviewer #2: Nicely written and appropriately presented with sufficient quality.

Reviewer #3: (No Response)

**Response 19:**

We thank the reviewers for their complements.

**Conclusions**

**Comment 20:**

Reviewer #1: The author expands the results of variables of their interest using other study results in discussion part, which sounds relevant to public health interest as the perception of epidemiological characteristics of COVID-19 and the effective strategy against it.

Limitations are well mentioned, including data collection bias, self-reporting bias and generalizability, immunity and hidden factors.

Conclusions highlights the importance of comprehensive healthcare strategy and characteristics of cases among the camp based on the analysis and findings.
**Response 20:**

We thank the reviewers for their complements.

**Comment 21:**

However, some interpretation from the examined results were not covered: for example, how do you understand the trend of IRR with some conditions? Please provide the readers with a clear explanation of how this finding does indicate.

**Response 21:**

Thank you for sharing your thoughts about the study. However, due to limited number of words of the main text, we discussed the most important changes in the incidences such as comparing incidence of the camp with the national figures, compare incidence and interventions within the villages of the camp, and between genders and modes of detections along the discussion. Sorry for not being clear about the explanation of the IRR. We made a few amendments to the text to clarify this point.

Reviewer #2: (No Response)

Reviewer #3: (No Response)

**Editorial and Data Presentation Modifications?**

**Comment 22:**

Reviewer #1: Some comments and points table- and figure-specific that I would recommend to modify:
*Add total number in each subcategories (Table 3).
*in Table 3 p-value: clarify which data to compare (maybe in the text)
*Add total number of cases per month in each figure (or separated table, etc.) such as Figure 4.
*Figure 2A: I think there is no point to exhibit no. of cases and incidence altogether unless there are difference in population between male and female.
*Figure 3: Consider the graph into cumulative number.
*Figure 3C: vaccination figure is missing.

**Response 22:**

1- Total number in subcategories is added.
2- We identified that Table 3 compares the case severity with the demographics and comorbidities groups, such as age and sex. We have clarified this in the text and the table.
3- The total number of monthly cases is presented in Figure 1 and figure 4. We cannot add the total number of cases in the other figure as the y-axis scales will be very long while the actual intent of plotting other variable will not be clear as it is in the current presentation.
4- We have deleted the number of cases for both genders in Figure 2A.
5- In Figure 3, the cumulative number was not added for better clarity of the graphs,
6- However, based on your suggestion, we added the missing Figure 3c, with the cumulative number for the vaccinated people, as it might provide an insight into that context.

Reviewer #2: (No Response)

Reviewer #3: (No Response)

**Summary and General Comments**

**Comment 23:**

Reviewer #1: As a result, I would recommend for major revision, as some of the premises in this analysis were missing, and the findings are dispersed, the conclusion is not clear, condition would not be well controlled. Methodologies in the analysis could be revised and implemented better.
Please consider the recurrent cases and re-do the analysis, considering the correlation. Also, if the authors would like to mention about monthly IRR change, describe more about linearity and their assumption, findings and its discussions (practical indication, etc.) so that the readers could understand better.

**Response 23:**

We sincerely thank the reviewer for their detailed feedback, which has greatly contributed to improving the quality and clarity of our manuscript. In response to the points raised, we have carefully considered and addressed each issue as follows:

- We clarified how recurrent cases were treated in the analysis, emphasizing their inclusion to capture the dynamics of different COVID-19 waves. These cases were treated as new infections when occurring in separate waves, given the distinct characteristics of each wave driven by emerging variants. This approach was explicitly explained in the methods and results sections to ensure transparency.
- We provided a more detailed explanation of how monthly IRR changes were calculated using Poisson regression, without assuming linearity. The manuscript now includes explicit descriptions of the methodology, the rationale behind using IRR, and the interpretation of these findings in the context of the pandemic's wave-like progression. Practical implications of these trends were also discussed to improve the readers' understanding.
- We revised the statistical analysis and results sections to address the reviewer’s concerns about dispersed findings and unclear methodologies. This includes adding footnotes to tables, clarifying comparisons, and ensuring that all methods are described in a way that aligns with the study objectives

Thank you for your valuable comments and contribution to this study, your effort is much appreciated. For further clarification, kindly refer to the previous sections for an in-depth discussion of each mentioned point separately.

**Comment 24:**

Reviewer #2: Introduction section should address -
84 – 96: Generally, lacks description of evidences from existing literature, specially in other similar settings.

**Response 24:**

We thank the reviewer for this comment. While investigations on COVID-19 incidence in camp populations remain limited, we have added relevant examples from both local and international contexts to provide a broader perspective.

*“A retrospective study was conducted in Greece to estimate the incidence and outbreaks of COVID-19 ‎among resident asylum seekers and refugees in camps across 9 months period. The results indicated an ‎incidence of 1758- 2052 per 100,000 in those communities, which was almost three times higher than ‎the general population of Greece.‎‎ In Jordanian context, only one study investigated the incidence among Zaatari and Azraq camps and found that the incidence rates were lower in the camps ‎compared to the general population of the country.8 They suggested that follow-up research on COVID-19, ‎investigating the trend of infection rate over time, the appropriateness of mitigation and public health ‎strategies, and the effect on the healthcare system, is paramount to understand the case in such ‎humanitarian settings. However, this previous study covered a limited period, namely during the first ‎and second waves of alpha and beta variants, prior to the distribution of COVID-19 vaccines in the ‎camps”.*

**Comment 25:**
96: How evaluating preparedness and response strategy is relevant this particular epidemiological study?

**Response 25:**

We thank the reviewer for this important question. Evaluating preparedness and response strategies is highly relevant to this epidemiological study as these measures directly influenced the spread, detection, and management of COVID-19 cases in the Azraq camp. During the pandemic, the International Medical Corps (IMC), under the guidance of the National Defence Law and the United Nations High Commissioner for Refugees (UNHCR), implemented a series of infection prevention and control measures aimed at mitigating the spread of the virus within this vulnerable population. We added the following the introduction section:

*“During the COVID-19 pandemic, IMC implemented several strategies in aspect of infection prevention ‎and control measures that were mandated by the National Defence Law and the United Nations High ‎Commissioner for Refugees (UNHCR). These strategies ensured conducting COVID-19 testing and ‎case management and promoting vaccination campaigns to mitigate the spread of COVID-19.^5, 6^ ‎Implemented measures involved temperature screening at entry points of the camp and hospitals, ‎active surveillance and contact tracing, social distancing and gatherings prevention, direct contact ‎isolation, strict follow up, preparing and upgrading isolation areas, as well as ensuring good practices ‎in providing healthcare services and providing personal protection equipment. Jordan's efforts at the ‎national and local levels, including the activation of the Defence Law and collaboration with health ‎authorities, aimed to combat the spread of COVID-19.^7^”*

**Comment 26:**
98 – 106: Rationale of the research is not clear. Why it is essential to investigate the epidemiology of COVID-19 in this particular setting? How it will help global community?

**Response 26:**

Further explanation about the necessity of conducting such a study is mentioned in the introduction.

*“Understanding and investigating the incidence of infections in humanitarian settings is critical to ‎control disease. Exploring incidence in this context could help the global community to grant equitable ‎access to healthcare and resources for the refugees' communities, and governments and authorities to ‎legislate appropriate strategies and policies to control future crisis in those communities. However, ‎there’s scarcity of what is known about the incidence of the COVID-19 in camp population in Jordan. ‎The lack of such knowledge restricts mitigating the impact of future pandemic and suggesting ‎appropriate intervention.“*

**Comment 27:**

Discussion section:
Please include some sorts of comparison with similar other settings with possible explanations of difference.

Generally, the study provides useful insight.

**Response 27:**

We thank the reviewer for this important suggestion. Starting from line 329, the discussion includes comparisons between our findings and results from local and similar settings, focusing on refugee camps and other humanitarian environments. For example, we compared the incidence rates and public health interventions observed in Azraq camp with those reported in studies from refugee settings in Greece and other low-resource environments.

However, we acknowledge that the scope of our discussion is limited by the scarcity of studies specifically investigating COVID-19 epidemiology in refugee camps. This gap in the literature constrains the depth of comparison and highlights the need for more research in this area to better understand the unique dynamics of disease transmission and public health interventions in such settings.

**Comment 28:**

Reviewer #3: The structure of the article is logical, with a clear progression from introduction to methods, findings, and conclusions. The tone is professional and objective.
While the article is comprehensive, it could benefit from more detailed discussions on the specific healthcare interventions implemented and their effectiveness in the camp.
To enhance the article, the author could include a more in-depth analysis of the specific public health strategies that were successfully implemented during the pandemic in the camp.

**Response 28:**

We sincerely thank the reviewer for their positive feedback, thoughtful comments, and constructive suggestions. In response to the suggestion for a more detailed discussion on healthcare interventions, we have expanded the manuscript to include a comprehensive analysis of the specific public health measures implemented in the Azraq camp during the COVID-19 pandemic. These measures, including active surveillance, contact tracing, vaccination campaigns, isolation protocols, and the provision of personal protective equipment, were instrumental in mitigating the spread of the virus.

We have also contextualized these interventions by comparing them to similar measures in other refugee settings, highlighting both their successes and limitations. This expanded discussion assesses how the implemented strategies contributed to the observed epidemiological trends in the camp and evaluates their broader implications for pandemic management in humanitarian contexts.

**Comment 29:**

Additionally, more qualitative insights from residents could provide a deeper understanding of their experiences and challenges.

**Response 29:**

We thank the reviewer for this insightful suggestion. We fully agree that incorporating qualitative insights from residents could provide a deeper understanding of their experiences, challenges, and perceptions of the public health interventions implemented during the pandemic. However, due to the retrospective design and the quantitative nature of the current study, collecting and analyzing such qualitative data was beyond the scope of our research. Additionally, the data available for this study were collected as part of routine public health measures, which focused on epidemiological and clinical variables, limiting the opportunity to explore residents' subjective experiences.

Recognizing the value of qualitative research, we have included a recommendation in the manuscript for future studies to address this important gap as follows:

*“Further, this study did not explore the psychological impacts of the pandemic on refugees, the long-term implications of COVID-19 on their health and well-being, or more qualitative insights from residents which could provide a deeper understanding of their experiences and ‎challenges‎. Further research is needed to explore these gaps using an appropriate research design”.*

**Comment 30:**

Strengths include a robust sample size, comprehensive data collection, and a clear methodology. However, a potential weakness may be the reliance on data from one camp, which could limit the generalizability of the findings to other refugee settings.

**Response 30:**

We thank the reviewer for acknowledging the strengths of our study. We also agree that relying on data from a single camp may limit the generalizability of the findings to other refugee settings. This was a deliberate focus of our study, as we aimed to provide an in-depth analysis of COVID-19 epidemiology in Azraq camp as a distinct case study, highlighting its specific characteristics and the effectiveness of its public health strategies. Furthermore, the findings can serve as a basis for comparative studies and inform future research exploring broader trends across multiple refugee settings. These considerations underscore the importance of tailoring public health strategies to the unique conditions of each camp while recognizing common challenges in resource-constrained environments. To address this potential limitation, we have acknowledged in the limitations section

**Comment 31:**

The article does not deeply explore the psychological impacts of the pandemic on refugees or the long-term implications of COVID-19 on their health and wellbeing.

Additionally, it could have addressed the challenges of implementing public health measures in such settings more comprehensively.

**Response 31:**

We agree with you, those are very insightful suggestions to consider. However, the data were collected prospectively at that time, so there is no sufficient data to explore the psychological impact on their well-being. Also, studying the psychological impact and the challenges of implementing public health measures were out of the scope of the objectives of our study.

*“Further, this study did not explore the psychological impacts of the pandemic on refugees, the long-term implications of COVID-19 on their health and well-being, or more qualitative insights from residents which could provide a deeper understanding of their experiences and ‎challenges‎. Further research is needed to explore these gaps using an appropriate research design”.*

We sincerely thank the reviewers and editors for their thorough and constructive feedback, which has significantly enhanced the quality and depth of our manuscript. In response to the comments, we have clarified methodological approaches, including the treatment of recurrent cases, IRR calculations, and risk factor analyses. We expanded discussions on public health interventions implemented in the Azraq camp, comparing them to similar settings, and emphasized their implications for managing pandemics in resource-constrained environments.

Additionally, we acknowledged limitations such as the reliance on data from a single camp and the absence of qualitative insights, and we recommended future research directions to address these gaps, including exploring the psychological impacts and long-term health implications of COVID-19 on refugees.

These revisions aim to strengthen the manuscript's contribution to global public health and its relevance to humanitarian settings. We are confident that the updated manuscript addresses all the reviewers' concerns and provides a comprehensive and impactful analysis.

Best regards,

Ahmad Alrawashdeh, PhD

Department of Allied Medical Science

Faculty of Applied Medical Sciences

Jordan University of Science and Technology

Irbid 22110, Jordan

Tel: +962 799864033

E-mail: aaalrawashdeh@just.edu.jo
